# Determinants of Undernutrition among Adult Tuberculosis Patients Receiving Treatment in Public Health Institutions in Shashemane Town, Southern Ethiopia

**DOI:** 10.1155/2021/4218023

**Published:** 2021-07-13

**Authors:** Adane Tesfaye Anbese, Gudina Egeta, Frehiwot Mesfin, Abinet Arega Sadore

**Affiliations:** ^1^Department of Public Health, School of Public Health, College of Medicine and Health Science, Dilla University, Dilla, Ethiopia; ^2^Department of Human Nutrition, College of Health and Medical Science, Haramaya University, Haramaya, Ethiopia; ^3^School of Public Health, College of Medicine and Health Sciences, Wachemo University, Hosaena, Ethiopia

## Abstract

**Background:**

Undernutrition and tuberculosis are the major concerns of underdeveloped regions of the world. Tuberculosis makes undernutrition worse and undernutrition weakens immunity, thereby increasing the likelihood that latent tuberculosis will develop into active disease. Nevertheless, little has been understood about undernutrition among patients with infectious disease like tuberculosis in Ethiopia. This study was conducted to determine the magnitude of undernutrition and its determinants among tuberculosis patients in Shashemane public health institutions, Southern Ethiopia.

**Methods:**

An institution-based cross-sectional study was conducted in two public hospitals and ten health centers of Shashemane Town from March 12, to April 12, 2017, among 368 adult tuberculosis patients on treatment follow-up. Sociodemographic and socioeconomic characteristics and anthropometric data were collected. Data were entered into EpiData version 3.3 computer software and analyzed using SPSS version 20. Bivariable logistic regression analyses were done to assess the association between outcome variable at bivariate analysis, and multivariable logistic regression model was used to assess factors that were independently associated with undernutrition. Odds ratios along with 95% confidence interval (CI) were estimated to measure the strength of the association, and level of statistical significance was declared at *P* value ≤0.05.

**Result:**

The overall magnitude of undernutrition among adult tuberculosis patients in this study was 28.8% (95% CI = 0.25–0.34). Patients in the age group of forty-five and above ((AOR = 3.39, 95% CI = (1.6–7.18)), residents in rural area ((AOR = 1.95, 95% CI = (1.07–3.54)), those with problem with eating ((AOR = 2.361, 95% CI = (1.332–4.185)), and those who are not on food supplementation ((AOR = 2.21, 95% CI = (1.06–4.58)) were significantly at higher risk of undernutrition.

**Conclusion:**

The magnitude of undernutrition in the study setting was found to be significantly higher. Age greater than forty-five, living in rural area, and lack of nutritional care and support were identified as the factors associated with undernutrition. Thus, relevant actors should give attention to fast nutritional intervention together with standard therapeutic regimen in the management of pulmonary tuberculosis patients to curb their nutritional derangement.

## 1. Background

Undernutrition is a major public health issue in developing countries, including Sub-Saharan Africa (SSA) [1]. It can be caused by an illness that impairs nutrient intake and metabolism, or it can be caused by an insufficient intake of macronutrients, micronutrients, or both [1, 2]. Acute malnutrition occurs as a result of a brief or severe food shortage or infections that cause fat loss and skeletal muscle wasting [3]. Undernutrition has a variety of negative effects on health, including increased susceptibility to infectious diseases and worsening of existing diseases. Adults who are malnourished are less productive, less active mentally and physically, and more susceptible to other diseases [3].

Undernutrition and tuberculosis (TB) are key global health issues of considerable magnitude in most of the underdeveloped regions of the world. TB is one of the big three deadly public health threats, along with human immunodeficiency virus (HIV)/acquired immunodeficiency syndrome (AIDS) and malaria and causes 1.5 million deaths every year. Undernutrition increases the risk of TB and in turn TB could lead to undernutrition [3] and makes people more susceptible to the development of active TB, which in turn contributes to the development of undernutrition. This vicious circle not only can impact individuals but also can easily transfer to their families and their communities [3]. TB is probably associated with more severe form of undernutrition than any other chronic illnesses. TB makes undernutrition worse and undernutrition weakens immunity, thereby increasing the likelihood that latent TB will be developed into active disease [4].

Many deaths are caused by malnutrition, which, if left untreated, overwhelms normal physical and cognitive development [3]. Tuberculosis (TB) and malnutrition have been included in the Millennium Development Goals (MDGs) and, more recently, in the Sustainable Development Goals (SDGs) and have received global attention [5]. Regarding their relationship, it is simply understood that undernutrition and TB have a synergistic relationship that has a negative impact on one another. However, researchers have attempted to apply this understanding in studying the relationship between tuberculosis and malnutrition, with conflicting results [3, 5].

Few studies revealed that the magnitude of undernutrition among TB patients ranged from 52 to 90% in low-income countries other than SSA countries [6–9], 43–78% in SSA [10–13] and 27.2% to 84.4%, [14–19] in Ethiopia.

The causes of undernutrition are multifaceted ranging from basic to proximate ones. Accordingly, food insecurity [20], low socioeconomic status [13], lack of formal education, receiving food supplements during TB treatment [21–23], unsanitary surrounding [24], lack of employment, and HIV/TB coinfection [7, 10, 25] were found to be some factors associated with undernutrition among TB patients in some developing world including SSA countries. Similarly, there was scanty information on factors associated with undernutrition among the study population in Ethiopia, where lack of formal education was identified to influence nutritional status of TB patients [16, 26].

The Ethiopian government has implemented TB control programs like “End TB” with the aim of reducing deaths attributed to TB few years back. Regardless of the efforts made to respond to nutritional status of patients suffering from infectious disease like TB and HIV/AIDS in Ethiopia, many patients have been died from the double burden of undernutrition and such diseases which requires further investigation of the magnitude of the problem and its associated factors.

However, little has been understood about the magnitude of undernutrition due to infectious diseases particularly TB and associated factors in low-income countries including Ethiopia. Therefore, this study was aimed to determine the magnitude of undernutrition and associated factors among adult tuberculosis patients in public health facilities of the study setting in an attempt to help specific actors to design appropriate interventions in the management of TB superimposed with undernutrition based on the study results.

## 2. Materials and Methods

Institutional based cross-sectional study was used among adult TB patients who were on follow-up in two government hospitals and ten health centers in Shashemane during data collection period from March 1, 2017, to April 1, 2017. The study was conducted in Shashamane Town, one of the towns in Oromia national region, state in West Arsi Zone. Shashamane is located at south of Addis Ababa about 250 km. The town lies roughly 7° 08′ 51″N to 7° 18′ 19″N latitude and 38° 32′ 43″E 38° 41′ 07″E longitude. The town is situated at the upper greatest east African rift valley.

It has subtropical middle land climatic zone. There are two hospitals and ten health stations in the town, which are owned by the government and one private hospital and 48 private clinics. Agriculture is the likelihood of the majority of the study population [27].

### 2.1. Source and Study Population

The source populations were all TB patients who were on follow-up in TB clinics in Shashamane, whereas study population was all adult TB patients who were on follow-up in two government hospitals in Shashamane (i.e., Shashemene Referral Hospital and Melkaoda Hospital) and ten health centers, namely, Abosto HC, Bulchana HC, Fursa HC, Karabate HC, Faji HC, Jigesa HC, cebbe, Aradano, Toga, and Kuyera HCs TB clinics.

### 2.2. Sample Size and Sampling Procedure

The sample size was computed by taking into account the magnitude of undernutrition among adult TB patients and associated factors from previous literature using a single population proportion formula and two population proportion formulas, respectively:

(1) $$\begin{array}{c} n=\frac{z{\left(\delta /2\right)}^{2}p\left(1-p\right)}{d^{2}}, \end{array}$$

where *Z*_*α*/2_ = at 95% CI, which is equal to 1.96.

The sample size was estimated for the former formula with the following assumptions: prevalence of undernutrition among TB patients to be 27.15% [17], 95% confidence level to be 1.96, degree of precision (d) to be 0.05, and 10% for nonresponse yielding the sample size of 332. On the other hand, TB patient's HIV sero-status [16] was considered to answer the he objective designed to identify factors associated with undernutrition among the study population using the aforementioned formula with the following assumptions: 95% confidence level to be 1.96, power of 80%, and proportion of TB patients who were undernourished among those who were HIV positive to be 77%; proportion of TB patients who were under nourished among those who were HIV negative to be 54% and 10% for nonresponse which yielded the sample size of 321. However, regardless of the computed sample sizes for magnitude of an outcome of interest and associated factors, we considered all 368 adult TB patients who were on follow-up for TB treatment at all public health facilities and aged above 18 years during the study period to enhance the power of the study, except pregnant women, patients who were critically ill and unable to communicate during the interview and patients with physical disability since this could affect the quality of anthropometric measurements and may underestimate or overestimate the magnitude of undernutrition.

### 2.3. Data Collection Instruments and Procedure

The questionnaire was initially developed in English by reviewing available literatures including Ethiopian demographic and health survey questionnaire and then translated into Amharic and Afaan-Oromoo and back translated to English by another person who had good command of English and local languages to check for its consistency. Five health extension workers and two diploma nurses with previous relevant experience were recruited as data collectors and supervisors, respectively, and given an extensive training for two days. The questionnaire was pretested before the actual task of data collection elsewhere in a similar setting, and some modifications were made on study tools as needed.

Data on sociodemographic and dietary counselling were collected from using an interviewer administered pretested structured questionnaire. Anthropometric data such as weight and height were measured to the standard to minimize inter- and intraobserver variations, mainly technical error of measurements (TEM). The weight was measured using digital bath balance, Seca, which is a German model for weight measurement, was used. Portable standing scale was calibrated on regular basis against known weight and recorded to the nearest 0.1 kg. A height measuring board height in cm was marked on a wall with a help of a measuring tape. All study subjects were measured against the wall without footwear and with heels together and their heads positioned and eyes looking straight ahead (Frankfurt plane) so that the line of vision was perpendicular to the body and wood scales were brought down to the top-most point on the head. The height was recorded to the nearest 0.1 cm. Review of records was made for type and severity of the disease and HIV/AIDS status. History of last menstruation was sought to know pregnancy status women study participants in addition to pregnancy test.

### 2.4. Operational Definition

#### 2.4.1. Undernutrition: Body Mass Index < 18.5 [13]

BMI 17 kg/m^2^–18.5 kg/m^2^ was mild undernutrition, moderate undernutrition was BMI < 17 kg/m^2^, and severe undernutrition as BMI < 16 kg/m^2^ [13]:

  Dietary counselling is a process by which a health professional with special training in nutrition helps people make healthy food choices and form healthy eating habits. Functional status is the ability to carry on normal daily activities.
  Working is being able to carry on normal daily activities and no special care needed.
  Not working is being unable to work but able to live at home and able to care for most of personal needs and requires assistance at some times.
  Bed ridden is being unable to care for self, require institutional, or hospital care.
  Nutritional care and support are having components such as nutrition education in health facilities, water, hygiene, or food safety interventions to prevent diarrhoea as well as provision of adequate quality/quantity of food and food aid by any organization.
  Problem with eating is having any of the following: mouth ulcer, nausea and/or vomiting, poor appetite, or pain/difficulty in swallowing.

### 2.5. Data Processing and Analysis

Body mass index (BMI) was calculated by dividing weight (in kg) for height (in m^2^). It is underweight if < 18.5, normal if 18.5–24.9, overweight if 25–29, and obese if ≥ 30 kg/m^2^ (WHO, 2008). Sociodemographic and economic data were cleaned manually and then entered into the computer using Epi-Info version 3.3, and statistical analysis was made using SPSS version 20, whereas descriptive summary (frequency distribution, proportion, mean, and standard deviation) was used to summarize the variable. Continuous variables like age and income were first transformed into categorical variables before they were analyzed. First, frequency of all the variables in the questionnaire was determined. Secondly, cross-tabulation was done between important variables, and their significance was seen. Bivariate and multivariable logistic regression was done to assess the association of factors with undernutrition. Variables with a *P* value of less than 0.25 in the bivariate analysis were entered into the final model. By calculating odds ratios, their 95% confidence limits and *P* value less than or equal to 0.05 were taken as statistically significant. Important variables were entered and analyzed using multiple logistic regressions in order to control for confounding variables. Finally, the results of the study were presented using tables.

### 2.6. Ethical Consideration

Ethical clearance and permission were obtained from Institutional Health Research Ethics Review Committee (IHRERC), Haramaya University, and official letter was sought. The study participant was informed about the purpose of the study and the importance of their participation in the study, and then written consent was obtained. The study participants were informed that the information they give is confidential, and names of participants shall not be written on the questionnaire. No one had access to the information except those who works on the research, and the information is used for the purpose of research. Each participant was informed that his or her participation is voluntary to participate in the study. Health education was given to study participants, regarding the problem of study.

## 3. Results

### 3.1. Socioeconomic and Demographic Characteristics

Out of 374 study participants initially sampled in the study, 368 have participated, making a response rate of 98.3%. The mean (±SD) age of the study participants was 34.2 (±12.2) years with a minimum age of 18 years and maximum age of 70 years. The majority of the respondents were male (200), constituting 54.3%, and the majority, 242 (65.8%), of the family had greater than four family members with the mean and SD of (5 ± 2), including the respondent. Regarding the residence of the participants, 188 (51.1%) are from Urban. One hundred twenty-six (34.2%) of the participants were Orthodox Christian by religion followed by Muslim (30.4%) and Protestant (17.4%). Ethnically Oromo, Amhara, and Woliyta make 254 (69%), 74 (20.1%), and 26 (7.1%) of the participants, respectively. The majority of the participants are farmers 142 (38.6%) followed by merchant and daily laborer who constitutes 23.1% and 10.3%, respectively. The educational status of participants revealed that one hundred twenty-one (32.9%) can read and write, one hundred three (28%) are illiterate, and the rest, 39.2%, are in primary school or above. On average, 163 (44.3%) of the family earn less than 800 ETB per month (Table 1).

### 3.2. Magnitude of Undernutrition

In this study, the magnitude of undernutrition was found to be 28.8% (95% CI = 0.25–0.34). Of this, 17.4% have mild, 6% have moderate, and 5.4% have severe undernutrition. Furthermore, 65.5% had normal weight based on BMI classification.

### 3.3. General Health Status

Of the 368 respondents, 182 (49.5%) did not get dietary counselling/advise by their treating health personnel. 92 (25%) received food supplement. Close to 39% of study participants have problem with eating. Among these, 83 (58.3%) have poor appetite and 36 (25.4%) have nausea or vomiting. 182 (49.5%) of study participants have other illnesses. Among these, 104 (57.1) have febrile illnesses and 32 (17.6%) have diarrheal diseases, during the last two months. Regarding the type of TB, 276 (75%) have pulmonary TB. 47 (12.8%) had previous history of TB treatment. 35 (9.5%) of study participants are HIV positive (Table 2).

### 3.4. Determinants of Undernutrition among TB Patients

Being in the age group of forty-five and above (AOR = 3.39, 95% CI = 1.6–7.18), rural residence (AOR = 1.95, 95% CI = 1.07–3.54), average household monthly income (AOR = 3.4, 95% CI = 1.154–10.22) having problems with eating (AOR = 2.361, 95% CI = 1.332–4.185), and lack of provision of food supplement (AOR = 2.21, 95% CI = 1.06–4.58) were determinants of undernutrition among adult TB patients (Table 3).

## 4. Discussion

This study has attempted to identify the magnitude of undernutrition and its predictors among adult TB patients in public health facilities of Shashemane town. Accordingly, the magnitude of undernutrition was found to be 28.8%. Moreover, age of participant, average household monthly income, current place of residence, eating problem, and provision of nutrition support were factors significantly associated with undernutrition among adult TB patients.

The magnitude was much lower than in studies conducted in Amritsar, India (69.2 percent) [6], Timor and Rote Islands (87 percent) [9], Malawi (59 percent) [12], Kenya (43 percent) [10], and Limbe and Buea Regional Hospitals, Cameroon (78.21). [11]. According to Ethiopian studies, the magnitude of undernutrition in this study was still lower than in studies conducted in Ambo, West Ethiopia (63.5 percent) [14], Metema (51 percent) [15], and Gamo Goffa Zone, South Ethiopia (50 percent) [19] but higher than in studies conducted in Addis Ababa, Ethiopia (27.15 percent) [17]. The difference may be explained by differences in the socioeconomic status, study area, lifestyle, feeding pattern and economic status of the countries, and sample size of the study. Moreover, in some of these studies, the BMI of the participants was taken at the time of diagnosis before starting anti-TB medications when patients have not recovered from their illness and were at the state of critical undernourishment which could overestimate the expected level of undernutrition.

Undernutrition among the study population can have different effects; it leads to poor outcome of the TB treatment, because undernourished TB patients have difficulty of drug metabolism; it affects productivity at personal, family, and country level. Aggravation of the disease and death are other consequences of undernutrition.

This study revealed that age of participants is strongly associated with undernutrition; the most affected group was 41 years and above. Study participants in the age group of 41 and above are three times more likely to be undernourished than those in age group of 18–24. This is not consistent with the study done in Dehradun, India, which revealed opposite finding [21]. The high magnitude of undernutrition in the elderly population in this study could be due to different socioeconomic status of the populations. Moreover, the lower metabolism, lower gastrointestinal function, and less tasting ability in old age can lead to undernutrition [3].

This study revealed that the place of residence was strongly associated with undernutrition. Adult TB patients living in rural kebeles were more likely to be undernourished than those living in urban kebeles. This could be due to the cumulative effect of series of less favourable socioeconomic conditions which in turn lead to less access to healthcare, balanced nutrition, safe water, and sanitation in rural compared to urban areas. This finding was not in agreement with study conducted in Ambajogai, India, which showed opposite finding [24].

Household monthly income had showed significant association with undernutrition. The odds of undernourished among TB patients increase when household monthly income is found to be below 800 Birr per month. This shows that the household monthly income was inversely related to undernutrition. This finding is in line with the other study conducted in Ghana [13], the reason for undernutrition being higher among those households with lower monthly income may be due to their lower purchasing capacity for food and unavailability of hygienic and healthy living environment among them.

Tuberculosis is associated with feeding problems, which have a direct impact on the patient's food intake. This study discovered that eating disorders, specifically loss of appetite, nausea, and vomiting, have a significant association with undernutrition. Adult TB patients who have eating problems have twice the magnitude of undernutrition as those who do not have eating problems. However, the findings of this study contradicted those of a study conducted in public health institutions in Addis Ababa, Ethiopia [27], which could be due to the difference in duration since starting antituberculosis medications, because the Addis Ababa study only included TB patients who were taking medications for up to two months, which was the expected time for the patients to start recovering from the disease in whom there are fewer episodes of loss of appetite, nausea, and vomiting.

Food and nutrition support breaks the vicious cycle of undernutrition and infection through two key mechanisms. Food and nutrition activities support nutritional stabilization and recovery, resulting in increased immune system strength, faster sputum clearance, and faster weight gain. Food and nutrition activities also support access and adherence to treatment. This study has revealed the provision of nutrition support significantly associated with undernutrition. Adult TB patients who were not provided with food and nutrition support are two times more likely to be undernourished than those who were provided. However, a study done in Addis Ababa, Ethiopia, revealed no statistically significant difference in risk of undernutrition with provision of nutrition support [27]. This difference could be explained by differences in the socioeconomic status of the two populations.

### 4.1. Limitation of the Study

Causal inferences can never be drawn out of the findings since the study is a cross-sectional one. Wealth index could be a better indicator of socioeconomic status, but average monthly income was used by this study. The TB treatment could improve nutrition status of TB patients, and this may underestimate magnitude of undernutrition among TB patients in this study because data were collected in TB patients during follow-up period time. During the interview, there may be a social desirability bias and recall bias by participants. Another limitation of this study is that we did not consider special eating style and pattern of study subjects.

## 5. Conclusion

The magnitude of undernutrition among adult TB patients was high with the majority of them having a mild form. Age greater than forty years, living in rural kebeles, earning less than 800 Birr per month, problem with eating, and lack of nutritional care and support were all positively associated with undernutrition. Based on the findings of this study, the following recommendations are forwarded: regional health office should incorporate nutrition supplementation and support as integral component of TB treatment and improve household income/economy through broadening microfinance institution or other development activities especially to rural community, so that dietary intake will be improved. It is recommended that the Woreda Health Office implement regular undernutrition screening program for TB patients based on BMI, and health extension worker should work hard on nutritional care and support. Finally, as this is an institution-based study, further community-based studies are recommended for researchers to identify risk factors for undernutrition.

## Figures and Tables

**Table 1:** Socioeconomic and demographic characteristics of TB patients in government health institutions of Shashemane, Ethiopia, 2017 (*n* = 368).

Variable		Frequency	Percent
Age in years	18–26	85	23.5
	27–35	59	16
	36–44	99	26.5
	>45	125	34

Sex	Male	200	54.3
	Female	168	45.7

Marital status	Married	245	66.6
	Single	113	30.7
	Widowed	5	1.4
	Separated	5	1.4

Family size	<4	126	34.2
	≥4	242	65.8

Religion	Orthodox Christian	147	39.9
	Protestant	78	21.2
	Catholic	21	5.7
	Traditional	2	0.5
	Muslim	120	32.6

Residence	Urban	188	51.1
	Rural	180	48.9

Occupational status	Farmer	142	38.6
	Merchant	85	23.1
	Daily labourer	38	10.3
	Government employer	33	9
	Housewife	32	8.7
	Student	38	10.2

Ethnicity	Oromo	254	69
	Amhara	74	20.1
	Tigrai	26	7.1
	Woliyta	10	2.7
	Other^*∗*^	4	1.1

Educational status have no formal education		103	28
	Can read and write	121	32.9
	Primary school	76	20.7
	Secondary school	58	15.8
	Above secondary school	10	2.7

*Average household income per month*			
<800		163	44.3
801–1500		163	44.3
>1500		42	11.4

^*∗*^Silte, Gurage, and Kembata.

**Table 2:** General health status of adult TB patients in government health facilities of Shashemane, Ethiopia, 2017 (*n* = 368).

Variable	Frequency	Percent
*Provision of food supplement*		
Yes	92	25
No	276	75
*Dietary counselling*		
Yes	186	50.5
No	182	49.5
*Problem with eating*		
Yes	144	39.1
No	224	60.9
*Type of eating problem*		
Mouth ulcer	9	6.3
Poor appetite	83	58
Nausea or vomiting	37	25.9
Pain or difficulty of swallowing	14	9.8
*Other illnesses*		
Yes	182	49.5
No	186	50.5
*Type of other illnesses*		
Diarrheal disease	32	17.6
Febrile illness	104	57.1
Hypertension	25	13.7
Diabetes	16	8.8
Cancer	2	1
Others^*∗∗∗*^	3	1.6
*Type of* TB		
Pulmonary TB	276	75
Extrapulmonary TB	92	25
TB treatment		
New case	321	87.2
Previous history of TB treatment	47	12.8
HIV/AIDS status		
Positive	35	9.5
Negative	333	90.5

^*∗∗∗*^Fever, urinary tract problem, and headache.

**Table 3:** Factors associated with undernutrition among adult TB patients in government health institutions of Shashemane, Ethiopia, April 2017 (*n* = 368).

Variables		Undernutrition		COR (95% CI)	AOR (95% CI)
		Yes	No		
Age	18–26	15 (14)	72 (27.5)	1	1
	27–35	7 (6.5)	52 (20)	0.738 (0.292–1.870)	0.879 (0.313–2.469)
	36–44	20 (18.9)	77 (29.5)		1.05 (0.46–2.4)
	>45	65 (60.7)	60 (23)	1.247 (0.593–2.62)	3.387 (1.597–7.181)^*∗∗∗*^
				5.2 (2.65–9.89)	

Family size	≤4	26 (24.3)	100 (38.3)	1	1
	>4	81 (75.7)	161 (61.7)	0.51 (0.31–0.853)	1.418 (0.77–2.6)

Residence	Rural	73 (68.2)	107 (40.9)	0.323 (0.199–0.156)	1.949 (1.08–3.538)^*∗*^
	Urban	34 (31.8)	154 (59.1)	1	1

Average income monthly	≤800	55 (51.4)	108 (41.3)	3 (1.225–7.765)	3.4 (1.154–10.22)^*∗∗*^
	8001–1500	46 (43)	117 (44.8)	2.359 (1.04–4.27)	2.67 (0.88–8)
	>1500	6 (5.6)	36 (13.8)	1	1

Nutrition care and support	Not given	94 (87.85)	181 (69.3)	3.214 (1.7–6.1)	2.209 (1.1–4.6)^*∗*^
	Given	13 (12.15)	80 (30.7)	1	1

Food supplement	No	92 (86)	184 (70.5)	2.57 (1.406–	1.1 (0.5–2.41)
	Yes	15 (14)	77 (29.5)	4.736)	1

Dietary counselling	No	67 (62.6)	115 (44)	2.1 (1.32–3.322)	1.467 (0.83–2.58)
	Yes	40 (37.3)	146 (56)	1	1

Problem with eating	Yes	64 (59.8)	80 (30.7)	3.36 (2.07–5.28)	2.36 (1.33–4.2)^*∗∗*^
	No	43 (40.2)	181 (69.3)	1	1

Presence of other illness	Yes	68 (63.5)	114 (43.7)	2.24 (1.4–3.55)	1.328 (0.744–2.37)
	No	39 (36.5)	147 (56.3)	1	1

Functional status	Working	45 (42)	129 (49.8)	1	1
	Not	41 (39.2)	17 (45.2)	1.013 (0.62–1.66)	0.83 (0.46–1.49)
	Working	21 (19.6)	13 (5)	4.63 (2.14–10.01)	2.25 (0.9–5.6
	Bed ridden				

History of previous TB treatment	No	85 (79.4)	236 (90.6)	1	1
	Yes	22 (20.6)	25 (9.6)	0.766 (0.432–1.356)	1.53 (0.695–3.34)

^*∗*^Significant at *P* ≤ 0.05, ^*∗∗*^significant at *P* ≤ 0.002, ^*∗∗∗*^significant at *P* ≤ 0.001. OR = odds ratio, AOR = adjusted odds ratio, and CI = confidence interval.

## Data Availability

The data are available from the authors upon reasonable request and with the permission of Shashemane Wereda Health Office.

## Abbreviations

AOR: Adjusted odds ratio
BMI: Body mass index
CI: Confidence interval
COR: Crude odds ratio
DOT: Direct observed treatment short course
FAO: Food agricultural organization
IRERC: Institutional research review committee
MDR: Multidrug resistance
MPH: Master of public health
NGO: Nongovernment organization
PEM: Protein energy malnutrition
PI: Principal investigator
PTB: Pulmonary tuberculosis
SES: Socioeconomic status
SPSS: Statistical Package for Social Science
TB: Tuberculosis
USAID: United States Agency for International Development
WFP: World Food Program
WHO: World Health Organization.
